# IRAMP: Investigation of Risk Assessment and Management Processes Using Staff Focus Groups

**DOI:** 10.1192/bjo.2024.255

**Published:** 2024-08-01

**Authors:** Kay Sunderland, Emma Drysdale, Brian Gillatt, Alan Mackenzie, Paula McCahon

**Affiliations:** NHS Greater Glasgow and Clyde, Glasgow, United Kingdom

## Abstract

**Aims:**

To investigate risk assessment and management processes across a health board in the context of the implementation of a new risk screening tool and policy through use of staff focus groups to identify how teams make decisions related to risk and gain an understanding of how the new CRAFT tool is used.

In mental health services, risk assessment and management are key responsibilities for clinical staff. A risk management tool that is structured and evidence-based aims to assist staff in managing risks including violence, self-harm, suicide and self-neglect.

It is not clear whether risk tools have clinical utility in influencing risk-related decision making and previous reviews within the health board indicated that risk policy was not being adhered to, prompting a review of the policy. Furthermore, policy recommends service user and carer collaboration with staff in all areas of mental health in Scotland but despite these recommendations there is little evidence to suggest they are routinely involved in risk assessment and management processes.

The present study is an opportunity to explore how teams think about and discuss risk management.

**Methods:**

A qualitative analysis was carried out of data from two staff focus groups. These groups were identified by contacting interested teams by email. Groups comprised clinical staff from different disciplines within the MDT including medical and nursing staff. Staff were questioned about their understanding of risk, thoughts regarding risk assessment and their experience of being trained in and using the CRAFT tool.

**Results:**

Themes emerging from the data indicate that staff felt the CRAFT had limited clinical utility or impact on their assessment of risk but may prove useful for communicating decisions about risk between staff and services. However, concerns were raised that the format of the tool made it difficult to complete and read, meaning that important information may not be adequately communicated. Staff reported feeling inadequately trained in the use of the CRAFT tool and felt there were inconsistencies in its use across the health board.

**Conclusion:**

Staff focus groups have identified challenges with the completion of the current CRAFT tool and expressed a need for better training in order to improve consistency of use across the health board. An update to the tool is due to be rolled out across the board in an effort to address these issues and improve risk assessment completion on the whole.

